# Increased Overlap between the Brain Areas Involved in Self-Other Distinction in Schizophrenia

**DOI:** 10.1371/journal.pone.0017500

**Published:** 2011-03-09

**Authors:** Renaud Jardri, Delphine Pins, Gilles Lafargue, Etienne Very, Aurély Ameller, Christine Delmaire, Pierre Thomas

**Affiliations:** 1 Laboratoire de Neurosciences Fonctionnelles et Pathologies, EA-4559, Université Lille Nord de France, Lille, France; 2 Service de Psychiatrie Infanto-Juvénile, Hôpital Fontan, CHRU de Lille, Lille, France; 3 Centre National de la Recherche Scientifique (CNRS), Paris, France; 4 Service d'Urgences Psychiatriques Universitaires, Hôpital Purpan, Toulouse, France; 5 Service de Psychiatrie, Hôpital Fontan, CHRU de Lille, Lille, France; 6 Service de Neuroradiologie, Hôpital Salengro, CHRU de Lille, Lille, France; North Lille University (USTL), France

## Abstract

Self-awareness impairments are frequently mentioned as being responsible for the positive symptoms of schizophrenia spectrum disorders. However, the neural correlates of self-other distinction in this pathology are still poorly understood. In the present study, we developed an fMRI procedure in order to examine self-other distinction during speech exchange situations. Fifteen subjects with schizophrenia were compared to 15 matched controls. The results revealed an increased overlap between the self and non-self cortical maps in schizophrenia, in the medial frontal and medial parietal cortices, as well as in the right middle temporal cortex and the right inferior parietal lobule. Moreover, these neural structures showed less BOLD amplitude differences between the self and non-self conditions in the patients. These activation patterns were judged to be independent of mirror-like properties, familiarity or body-ownership processing. Significantly, the increase in the right IPL signal was found to correlate positively with the severity of first-rank symptoms, and thus could be considered a “state-marker” of schizophrenia, whereas temporal and medial parieto-frontal differences appear to be “trait-markers” of the disease. Such an increased overlap between self and non-self cortical maps might be considered a neuro-physiological signature of the well established self-awareness impairment in people suffering from schizophrenia.

## Introduction

For centuries, free-will and self-consciousness have been striking subjects of interest in various sectors of society. Within this framework, distinguishing oneself from others appears to be crucial for social interactions. However, this ability is far from absolute, since it may be impaired in certain pathological conditions, such as in schizophrenia, in which patients regularly fail to identify their own actions or thoughts, by over-attributing them to external sources [1], [2]. In fact, a growing body of evidence suggests that the capacity to experience oneself as the agent of one's own actions (agency) might concur, along with the sense of body-ownership, with self-identification in general [3]. Importantly, in daily-life activities, we do not constantly think about the source of our perceptions, and it could be postulated that this moment-to-moment selfhood experience may be mainly linked to non-conscious cognitive processes, which only manifests itself when a situation requires it. One of the most influential models for understanding the nature of brain processes involved in distinguishing oneself from other is the central monitoring theory (CMT). In the CMT, the agent is determined by online monitoring of the degree of congruence between internally-generated predictions and peripheral signals, generated by an action [4], [5], [6]. Some neuroimaging studies in the field of visuo-motor agency suggest that the temporo-parietal junction (TPJ), and especially the inferior parietal lobule (IPL), plays a key role in disentangling the origin of sensory events [7], [8], [9].

However, the CMT is unable to account for agency without execution of an action, as in mental imagery, in which an action is represented by the motor system, without being executed [10], [11]. An alternative hypothesis particularly useful for resolving this problem is proposed by the shared-representation model (SRM). According to the SRM, the motor system simulates an action, by whomever the agent is (oneself or somebody else), and these two modalities of action representations share the same neural structures [12], [13]. This theory is supported by the existence of mirror neurons which were first shown in the Macaque monkey brain. Mirror neurons discharge during both execution and observation of an action [14], [15]. A Mirror Neuron System (MNS), similar to that of the monkey, has been found in humans and may be involved in many higher motor functions, from coding intended actions [16] to language processing [17]. Interestingly, the previously mentioned right IPL, has also been shown to be involved in the MNS [18], nevertheless separate areas for mirror responses and agency were identified within the parietal region [19]. This result is consistent with the idea that the MNS does not directly provide by itself, a representation of self. Consequently, the SRM assumes that the overlap between neural activations, resulting from both of these forms of action representation, is incomplete. Thus, the most suitable explanation to account for the differentiation between oneself and another agent, is that it occurs on the basis of non-overlapping areas [13], such as in the IPL.

Our team recently developed and validated an fMRI paradigm in healthy subjects, which allows shared representations to be examined during situations with speech exchange [17]. In order to suppress potential contaminations of the task by means of body-ownership sensations commonly observed in visuo-motor agency tasks [20], we assessed the neural substratum of self-other distinction in the language domain. We used two types of sensorimotor verbal conditions: covert speech production and passive listening to somebody else's voice. This procedure showed in healthy subjects that the self-other distinction relied on the activity modulation of a medial fronto-parietal network and the right IPL. While another sub-part of the IPL is recruited more during the encoding mirror response, we also checked that these areas were not activated simply because the subject recognized the voice of the person speaking [17].

As some authors proposed to directly relate language to nuclear symptoms of schizophrenia [21], it seems particularly important in this disorder to test the self/non-self distinction in the verbal domain. In the present study, we used the previously described procedure to investigate the neural substrates of the self-other distinction in schizophrenia. Schizophrenia is a particularly devastating disorder, in which thought insertions, delusions of alien control and auditory-verbal hallucinations constitute “first-rank symptoms”: FRS [22]. We have already mentioned that these symptoms may be related to an incapacity to recognize their own thoughts and actions as being internally generated, but instead attribute them to alien entities [23]. Interestingly, the temporo-parietal junction has been shown to be involved in this type of process in schizophrenia. Using a PET-scan, Spence et al. observed that schizophrenic patients experiencing delusions of control during testing, showed abnormally high activity in the parietal region, when making voluntary movements [2]. Using fMRI in a task that revealed any discordance between hand movements being performed by the subjects, and sensory feedback displayed on a computer screen [7], other authors have noted an abnormal relationship between the degree of control of the movements, and activity in the right IPL in patients with schizophrenia [24]. However the vast majority of experiments conducted in schizophrenia, only explored explicit self-other distinction, based on attribution judgment paradigms [24], [25], [26], [27], [28]. These studies were insufficient to clearly determine if external misattributions by patients result more from a perceptual experience disorder, or from an impairment of judgment itself, which would be more linked to a thought disorder [29]. In such a basic context, it seems important to test self-other distinction at a lower level of processing, that is to say when no explicit distinction between self and non-self is required. Our main hypothesis was that distinguishing between internally and externally-generated stimuli in schizophrenia would rely on a low-level impairment that might be related to an increased overlap between self/other neural representations. Our study would also make it possible to determine the exact role of the IPL in patients suffering from schizophrenia, during the self-other distinction process.

## Methods

This research was approved by the local ethical committee (Comité Consultatif de Protection des Personnes se prêtant à une Recherche Biomédicale de Lille, CCPPRB Lille, n°CP 06/52).

### Participants

30 right-handed participants, according to the Edinburgh laterality test [30], who spoke French as their first language, and who gave their written consent, were included in our study. They were split in two groups: 15 patients with a diagnosis of paranoid schizophrenia according to the DSM-IV-TR classification [31] and 15 matched controls. Exclusion criteria for the whole population were the presence of an axis II diagnosis or another axis I diagnosis, a neurological or an Ear, Nose and Throat disorder, or a history of substance or alcohol misuse. Patients with an IQ below normal range were also excluded (WAIS-R). The main medical, social and demographic characteristics of these subjects are summarized in ***Table 1***. All of the patients had received regular doses of antipsychotic medication for at least one month prior to testing. A quantitative assessment of the symptoms was performed using the PANSS [32] either one day before, or on the day of the fMRI scan. All of the patients presented with at least two FRS. Finally, even though some of them had experienced refractory hallucinations during the weeks before they were examined, none of them reported auditory or visual hallucinations during the fMRI scanning procedure.

**Table 1:** Demographic information of the participants included in the ‘implicit self-other distinction’ study (n = 30).

	Patients with schizophrenian = 15; mean +/− sd	Healthy controlsn = 15; mean +/− sd	Group comparison	Significance	Comments
Age (yr)	*30.8+/−7.6*	*30.1+/−6.6*	*Z = 0.23*	*p = 0.82*	*-*
Handedness-ratio (R/L)	*15/0*	*15/0*	*NA*	*NS*	*-*
Sex-ratio (M/F)	*13/2*	*10/5*	*Z = 1.60*	*p = 0.11*	*-*
Education (yrs from High school)	*2.4+/−1.8*	*5.9+/−1.6*	*Z = 3.41*	*p<0.01*	*introduced as a covariate in later statistical analyses*
Illness duration (yrs)	*11.2+/−5.9*	*NA*	*NA*	*NA*	*-*
Antipsychotic dosage (mg/day CPZ-Eq)	*683+/−462*	*NA*	*NA*	*NA*	*-*
PANSS total score	*82.8+/−14.6*	*NA*	*NA*	*NA*	*-*
PANSS positive subscale	*22.5+/−5.5*	*NA*	*NA*	*NA*	*-*

*The z-scores shown in this table come from the Wilcoxon non-parametric test; sd: standard deviation; ; Yrs: years; NA: not applicable; NS: not significant; M: male, F: female subjects; R: right; L: left; CPZ-Eq: daily therapeutic equivalent dose for the antipsychotic medications, using Chlorpromazine as a reference*.

### Stimuli

Three conditions with different stimuli were used: Self Generated Voice <SGV>, unfamiliar Externally-Generated Voices <EGV> (2 males, 2 females) and Reverse-Taped Voice <RTV>. During the sound recording, the speakers read Paul Eluard's poem “La ville de Paris renversée”. Such connected-speech-stimuli were chosen because they including both semantic and prosodic information, and not just words or phonemes [33]. Their voices were recorded using a digital recorder (*Tascam TEAC DA-P1*) with a sampling rate of 44.1 kHz, and a resolution of 16 bits. One of the unfamiliar voices was altered and played backwards <RTV>, which disrupted the language making it incomprehensible, but kept the same frequency and prosodical properties. This transformation allowed a stimulus that the subject was unable to reproduce himself, either by means of an object, such as a musical instrument, or by his voice to be used, since it is biomechanically impossible to join together the breathed or blown syllables of this artificially reversed voice. All of the stimuli lasted for a total of 21 seconds. The amplitude of the sound files was normalized to 96%, in order to obtain a comparable amplitude for the different recordings (75 dB SPL).

### Experimental procedure

The paradigm used in our study was designed to find the neural correlates of implicit self-other distinction in language. The participants had to perform the experiment inside the scanner. The subjects lay down with their eyes closed, wearing MR-compatible headphones, which transmitted sound stimuli, and attenuated the ambient noise of the scanner (a reduction of about 30 dB SPL) (*MRI Devices Corporation, USA*). The experiment used a block-design paradigm, consisting of an initial silent period lasting 1 minute, to allow the subjects to get used to the noise of the scanner, followed by an alternation of stimuli lasting 21 seconds, and a rest lasting 12 seconds. The alternating stimulus/rest cycle was repeated 8 times per condition. The different conditions were presented to the different subjects in a random order. The subjects were asked to repeat the poem to self and not move their mouth during the <SGV> condition, while listening to their own voice through the headphones at the same time. Artifacts caused by face and head movements were avoided by this covert speech procedure. During the <EGV> and <RTV> conditions, the subjects had to listen to the voices passively. The subjects were never informed about the origin of the voice heard (self or other), and a sound signal (500 msec) of a different frequency preceded the different stimuli. Moreover, giving the subjects the task to mentally repeat or pasively listen to, diverged them from making explicit self-other judgements. The implicit testing condition for self and other was achieved, since during the task, the subject had to covert speak after hearing a certain high-pitched sound signal, and was not required to use their ability to distinguish between their own voice and the somebody else's voice (judgment of attribution). Testing self-other distinction at an implicit level as in this study required that no behavioral measurements of the subjects' responses were made during scanning.

**Figure 1:**
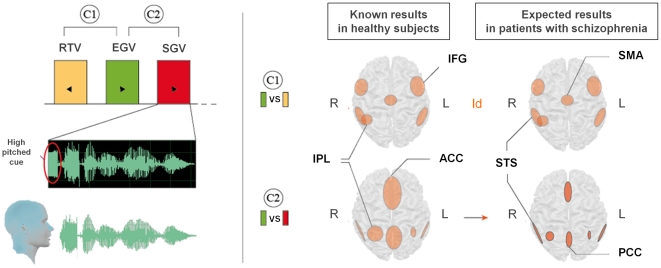
Diagram of the fMRI block-design with the expected results. In the upper left panel, a model of each experimental condition is shown: RTV (reverse-tape voice: yellow), EGV (externally-generated voice: green) and SGV (self-generated voice: red). Each block lasted 21 sec. and was repeated 8 times in a random order, each time being followed by a 12 sec. silent period. Part of the recorded voice spectrogram is shown in detail, as an example of the SGV condition, preceded by a 500 msec. high-pitched cue, which indicated that the subject had to mentally repeat what he was hearing (covert speech). The other conditions [EGV, SGV] were preceded by low-pitched cues, indicating that the subject should listen passively. Two contrast analyses were performed, and are shown in the right panel of the figure. C1 contrasted EGV and RTV, and revealing brain areas from the Mirror-Neuron-System. C2 contrasted EGV with SGV and revealed the cortical network involved in self-other distinction. The middle column shows known results from healthy subjects for C1 and C2 [17]. The right column lists the expected results in the patients with schizophrenia for the C1 and C2 contrasts. In our study, we hypothesized that in schizophrenics, there would be more overlap between the neural networks activated for representation of other or self (C2). This result cannot be explained by dysfunction in the mirror neuron system (C1). IFG: Inferior Frontal Gyrus/IPL: Inferior Parietal Lobule/ACC: Anterior Cingulate Cortex/PCC: Posterior Cingulate Cortex/STS: Superior Temporal Sulcus/SMA: Supplementary Motor Area/R-L: Right or left side of the brain, respectively.

Two control-situations were implemented outside the scanner in order to check that participants did not perform explicit judgments about the voices heard during scanning. First, the experiment included a preliminary training step for each subject, allowing us to ensure that the explicit task of covert speech was well understood and correctly performed. We did not use the subject's pre-recorded voice for the <SGV> condition during this training step. Second, a post-session interview was systematically realized to ensure that any of the participant performed attribution judgments during scanning. The full procedure is shown in ***Figure 1*** for the condition with a high-pitched initial signal (<SGV>).

### Data acquisition

Imaging was performed using a 1.5 Tesla MRI scanner (*Intera Achieva*, *Philips*, *The Netherlands*) with a SENSE-Head coil containing 8 elements. The T1-weighted anatomical sequence was a 3D multi-shot TFE with the following properties: 140 slices, thickness = 1.1 mm, FOV = 240 mm^2^, matrix = 256×256, TR = 8.2 msec, TE = 4 msec, flip angle = 8°, TFE factor = 192. The T2*-weighted functional sequence was a single-shot sensitivity-encoded echo planar imaging sequence (SENSE). A run of 280 volumes was obtained with a 30-slice Fast Fourrier Echo, thickness = 4 mm, FOV = 240 mm^2^, matrix = 64×64, TR = 3000 ms, TE = 70 ms, flip angle = 90°, SENSE factor g = 1.4.

### Data analysis

The functional data were pre-processed and analysed using *BrainVoyager QX v1.9* software (*Brain Innovation*, *The Netherlands*, *2008*). Images were pre-processed using slice scan time correction, 3D head motion correction, temporal high-pass filtering with 3 cycles/point, linear trend removal and 3D spatial smoothing, with a Gaussian filter of 5.0 mm. The anatomical data were submitted to an intensity unhomogeneity correction algorithm, resampled to 0.5 mm resolution and normalized in the stereotactic Talairach's space [34]. Head-tissue, subcortical structures and the cerebellum were then removed, to allow for advanced cortical segmentation processing. This segmentation was performed at the grey/white-matter and the grey-matter/cerebrospinal fluid boundaries, and each resulting hemisphere was submitted to a “bridge-removal” algorithm. For visualization of the statistical maps, the slice-based functional data were aligned on a high-quality 3D anatomical image. Finally, the cortical surface was reconstructed and inflated. *Cortex Based Alignment* was performed using curvature information, to improve anatomical inter-subject correspondence mapping beyond Talairach's transformation [35].

At a first level, *multisubject random effects analysis* was performed for each group, according to the *general linear model* [RFX-GLM] [36]. We defined a factorial design, in which the predictors of interest (<SGV>, <EGV> and <RTV>) and of no-interest (x,y,z motion parameters in translation and rotation) were derived by convolution of an appropriate box-car waveform, with a double-gamma haemodynamic response function. During second level analysis, we compared the two groups using a *repeated measure ANOVA model*, with the participants' group being defined as an inter-subject factor. Educational level was introduced as a covariate of no interest in this 2^nd^ level analysis. Since all subjects received the same stimuli, the conditions applied to the subjects in each group were defined as intra-subject factors (the [EGV – SGV] contrast for example). This bi-factorial ANOVA model was run over all of the voxels to obtain RFX maps. The statistical maps resulting from these first- and second-level analyses, were thresholded using the *false discovery rate* approach for multiple comparison correction [37]. To investigate with greater precision the spatial differences in the activated clusters between the groups for specific contrasts, we calculated *probabilistic functional maps* using the aligned surface maps previously produced. In order to achieve the macro-anatomical alignment of the gyri and sulci, the specified activation clusters of interest for each subject were mapped onto a common group space, prior to calculation. Finally, in the patient group, we performed *correlation analysis* between the quantitative functional activities in the regions of interest identified in the second-level analysis, and symptom severity, based on the PANSS total and subscale scores using the *SigmaPlot v10.0 software* (*Systat*, *USA 2007*). In this correlation analysis, the dosage of the antipsychotic drugs was introduced as a covariate of no interest.

## Results

The t-test, F-test peaks and the corresponding corrected p-values are shown in ***Tables 2*** ***and*** ***3***.

**Table 2:** Cortical areas involved in the identification of intelligible speech in healthy controls and patients with schizophrenia.

	BA	Side	t-values	p (corrected values)	Talairach coordinates
***Healthy controls***					
Pre-central gyrus	4	R+L	4.5	0.001	(-)44/-18/39
Medial frontal gyrus (SMA)	6	R+L	4.2	0.001	(-)3/-4/59
Insula	13	L	6.1	0.001	-43/8/5
Middle temporal gyrus	21	R+L	3.7	0.002	(-)51/-28/-10
Anterior cingulate gyrus	32	L	4.5	0.002	-3/32/29
Inferior parietal gyrus	40	R	5.9	0.001	47/-33/40
Inferior frontal gyrus (Broca's area)	44, 45	L	6.8	0.001	-46/18/16
***Patients with schizophrenia***					
Pre-central gyrus	4	R+L	4.1	0.001	(-)45/-17/37
Medial frontal gyrus (SMA)	6	R+L	4.2	0.001	(-)2/-4/56
Insula	13	L	5.7	0.001	-45/8/4
Middle temporal gyrus	21	R>L	4.1	0.001	(-)53/-27/-9
Anterior cingulate gyrus	32	L	4.6	0.002	-1/29/31
Inferior parietal gyrus	40	R	5.9	0.001	42/-34/43
Inferior frontal gyrus (Broca's area)	44, 45	R+L	6.5	0.001	-46/19/17

BA: Broadmann's areas; R/L: right or left side of the brain; SMA: supplementary motor area.

**Table 3:** Cortical areas involved in implicit self-other distinction in healthy controls and patients with schizophrenia.

	BA	Side	t-values	p (corrected values)	Talairach coordinates
***Healthy controls***					
*Post-central gyrus*	3	R	−8.5	0.002	(-)58/-16/26
*Pre-central gyrus*	4	L	−11.8	0.001	-57/-8/22
*Medial frontal gyrus*	6	R	−10.9	0.001	3/-5/56
*Medial frontal gyrus*	8	R+L	−7.9	0.003	(-)3/46/39
*Inferior frontal gyrus*	44	L	−7.8	0.003	-57/7/11
*Dentate nucleus (cerebellum)*	NA	R+L	−10.3	0.001	(-)19/-53/-33
*Caudate nucleus*	NA	L>R	−7.9	0.003	(-)17/-1/22
*Thalamus*	NA	R+L	−7.6	0.003	(-)10/-18/10
*Posterior cingulate gyrus*	23	R+L	8.0	0.002	(-)3/-54/20
*Posterior cingulate gyrus*	31	R+L	8.3	0.002	2/-54/26
*Anterior cingulate gyrus*	24	R+L	7.6	0.003	(-)3/32/12
*Anterior cingulate gyrus*	32	R+L	9.2	0.002	(-)3/34/18
*Superior temporal gyrus*	39	R+L	5.9	0.004	(-)47/-54/12
*Inferior parietal lobule*	40	R>L	7.8	0.003	44/-38/40
***Patients with schizophrenia***					
*Post-central gyrus*	3	R	−8.2	0.002	(-)59/-15/26
*Pre-central gyrus*	4	L	−10.5	0.001	-56/-7/23
*Medial frontal gyrus*	6	R	−10.7	0.001	2/-4/54
*Medial frontal gyrus*	8	R+L	−8.0	0.002	(-)4/41/43
*Inferior frontal gyrus*	44	L	−7.6	0.003	-58/6/13
*Dentate nucleus (cerebellum)*	NA	R+L	−9.9	0.002	(-)9/-53/-30
*Caudate nucleus*	NA	L>R	−8.0	0.002	(-)13/4/14
*Thalamus*	NA	R+L	−7.6	0.003	(-)9/-17/7
*Posterior cingulate gyrus*	23	R+L	9.8	0.002	(-)1/-56/21
*Posterior cingulate gyrus*	31	R+L	10.1	0.001	1/-51/32
*Anterior cingulate gyrus*	24	R+L	9.3	0.002	(-)2/33/12
*Anterior cingulate gyrus*	32	R+L	11.2	0.001	(-)1/32/20
*Superior temporal gyrus*	39	R+L	5.5	0.004	(-)47/-52/11
*Middle temporal gyrus*	39	R>L	5.9	0.004	(-)45/-59/22
*Inferior parietal lobule*	40	R>L	9.7	0.002	43/-39/38

BA: Brodmann's areas; R/L: right or left side of the brain; SMA: supplementary motor area.

### Behavioral data

All participants without exception performed successfully the training phase before the MRI-scanning for the explicit task (mentally repeat during <SGV>, listen during the other conditions). Moreover, post-session interviews revealed that no subjects from the patient and the control groups identified their own voices in the SGV condition, avoiding the possibility of explicit attribution judgments during the task.

### Activation network for intelligible speech

Intelligible language, tested by [EGV – RTV] contrast RFX analysis, showed bilateral activations in both groups, in brain regions which are part of the MNS (Cf. ***Table 2***). More precisely, activity was measured in Broca's area, the left insula, in the middle part of the precentral gyrus (bilaterally), the supplementary motor area (SMA), the middle temporal gyrus (MTG) and the right inferior parietal lobule (IPL). The second level of this particular contrast analysis, corrected for “education” disparities, found increased activity in the right part of the MNS network (including the middle temporal gyrus), for patients with schizophrenia compared to the matched controls. Note that no activity was found in the mirror-like areas with [RTV – EGV] reverse contrast analysis.

### Activation network for self-verbal agency

#### 1st level analysis

Implicit other-self differentiation was tested by [EGV – SGV] contrast RFX analysis in each group (Cf. ***Table 3***). As already shown for healthy subjects, this analysis showed three different haemodynamic patterns [17]. First of all areas, that are classically more activated in language production <SGV> than in the listening condition <EGV>, were identified in the cortical and sub-cortical regions. As expected, higher activity at the level of premotor and motor cortices with the production condition was found in each group. Then, brain areas that were more activated in the listening conditions than in speech production were grouped into two different clusters. The first cluster corresponded to the areas activated during the <EGV> condition, which were simply activated less, or stayed at the baseline level during the <SGV> condition: the middle temporal gyri (Brodmann's area BA 39), and the right IPL at the level of the supramarginalis gyrus (BA 40). The second cluster included the areas that presented no reactivity for the listening conditions, but were deactivated during speech production, with respect to the resting condition: the ventro-medial prefrontal cortex (BA 32, 33) and the posterior cingulate cortex (BA 23, 31).

#### 2nd level analysis

**Figure 2:**
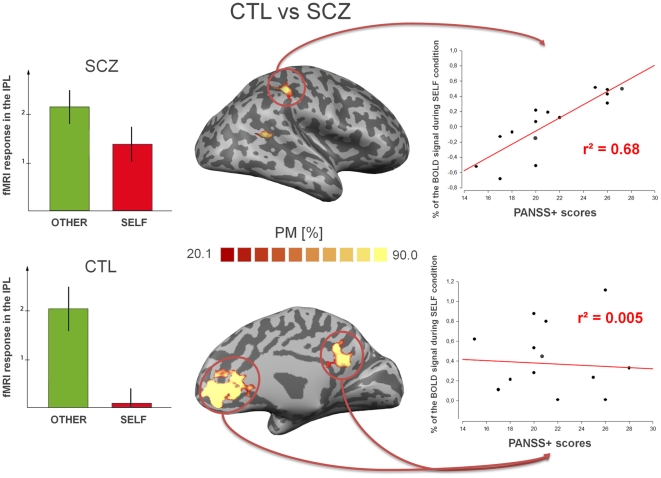
The self-other distinction network in patients with schizophrenia, compared with healthy controls. In the middle column, the red-to-yellow colour-code represents the relative percentage of subjects with greater activity in the [EGV – SGV] contrast, after we performed cortex-based normalization between the healthy matched controls (CTL) and the patients suffering from schizophrenia (SCZ). %PM: percentage of activity in the subjects included at a specific spatial location, in a range from 20 to 90 (spatial consistency). In the patients, the difference in the [EGV – SGV] contrast was significantly less marked than for the controls, which was also confirmed by the mean haemodynamic response of the fMRI signal in the right inferior parietal lobule (IPL). This is shown for each group in the left panel. The green bars represent the EGV condition, and the red bars show the SGV condition. In the right column, correlation analysis (adjusted for the dosage of the antipsychotic drugs) between the intensity of the fMRI signal in the self-other distinction network and the severity of the positive symptoms in schizophrenia (PANSS positive subscale) is shown.

Finally, repeated ANOVA measurements showed an interaction effect between the groups and the conditions within the parieto-temporo-frontal network when differentiating others from self. This second-level analysis, corrected for “education” variations, showed increased activity for this contrast for the healthy subjects, compared with the patients with schizophrenia in the right IPL (**F_28_ = 20.22**; **p<10^−4^**), the right MTG (**F_28_ = 21.80**; **p<10^−5^**) and in the medial frontal (**F_28_ = 22.93**; **p<10^−6^**) and medial parietal cortices (**F_28_ = 23.46**; **p<10^−4^**), in the anterior and posterior cingulate cortices respectively. As an illustration, the lowest difference of amplitude of the haemodynamic responses observed between externally generated voices and the self (EGV and SGV respectively) in the right IPL in the patients with schizophrenia compared with the healthy controls, results from an increased activity during the self condition (Cf. ***Figure 2***). In each group, probabilistic functional maps were then computed. These probabilistic maps confirmed up to 80% spatial consistency for the activity patterns in this neural network in each group, amongst all of the subjects tested, and good homogeneity for each experimental group. Within this network, we compared the surface extent of the clusters in the other-self contrast between the two groups using the following formula: ([voxels_EGV_−voxels_SGV_]/voxels_EGV_), and found significantly larger cluster sizes in the healthy subjects compared with the patients, in the right IPL (**t_28_ = −2.96**; **p<10^−3^**), the right MTG (**t_28_ = −2.46**; **p<10^−2^**), the medial frontal (**t_28_ = −3.67**; **p<10^−4^**) and medial parietal cortices (**t_28_ = −3.29**; **p<10^−3^**). These results all support a higher degree of overlap between self/non-self activations in schizophrenics compared with controls.

### Correlation with severity of the positive symptoms

Correlation analysis was performed in the parieto-temporo-frontal network, which has been previously identified. The activity level in the right IPL was initially found to correlate positively with the PANSS positive scores (**r^2^ = 0.68**; **p<10^−5^**), although no such correlation was found in the temporal cortex or the medial brain areas of this network (r^2^<10^−3^). Furthermore, no correlation was identified between the BOLD level in these areas and the other scores (negative, general or composite subscales, total PANSS score). After adjustment for the “dosage” factor, the analysis was repeated, but the response profile remained the same. The signal increase in the right IPL remained strongly correlated with the PANSS positive scores (**r^2^ = 0.65**; **p<10^−4^**), but no such link was found between the severity scores and the temporal or medial brain areas.

## Discussion

Using fMRI, we investigated the neural correlates of implicit self-other distinction in the verbal domain, in normal adults and patients suffering from schizophrenia. For both groups, other/self differentiation was accompanied with a high spatial consistency, by activation on the medial brain surface, of a neural network composed of the anterior and posterior cingulate cortices, coupled with right-sided structures, including the temporal cortex and the inferior parietal lobule (IPL). This result supports the idea that the schizophrenic patients use a comparable neural network to controls during social interaction, such as speech exchange situations. The involvement of these brain areas in implicit self-other distinction agrees with a meta-analysis showing IPL involvement in both explicit and low-level social cognitive processes [38]. Another key finding in our study is that the difference between non-self and self cortical maps was smaller in the patients suffering from schizophrenia than in the controls. Indeed, the patients recruited more overlapping brain maps for self and non-self, as demonstrated by the cluster-size calculation between the two groups in addition to the weakest BOLD amplitude differences between the self and non-self conditions in the patients (cf. *Figure 2*). Finally since antipsychotic drugs can potentially disturb BOLD measurements, the strength of our results was reinforced by the finding that the effect remained, even after adjustment for the dosage factor. Altogether, our data are compatible with the idea of selectively-impaired implicit self-other processing in schizophrenia, which may account for passivity phenomena, independently of any MNS disturbance [39], since no hypofunctioning of this mirror network was observed during the intelligible speech listening contrast in the patients, compared with the healthy controls. Such a small difference between self and other related activations in patients with schizophrenia could be interpreted by referring to the SRM [12], [13]. As already mentioned in the introduction, this model is supported by neuroimaging studies showing a partial overlap in cortical activations during three types of action: those produced by oneself, those entirely mentally simulated, or those observed in other people. In this way, discrimination between self and non-self may occur, on the basis of non-overlapping areas, by means of a neural ‘Who’ system [13]. In this setting, the pathological phenomenon of FRS in patients with schizophrenia, may result in self/non-self ambiguities, as suggested by Jeannerod [40].

However due to the results of the present experiment, different response profiles have to be distinguished within this network. On the one hand, no correlation with the severity of positive symptoms in the patients with schizophrenia was demonstrated for the medial parieto-frontal structures. This leads us to propose that dysfunctions of this medial network would more traduce a state-independent pattern, i.e. whatever the severity of actual symptoms is. A finest examination of the developmental trajectory of these brain areas also supports a ‘trait-marker’ hypothesis. First, the perigenual portion of the ACC, part of the medial parieto-frontal network recruited during self-other distinction, has a more progressive growth than caudal or dorsal portions [41]. In the same line of thoughts, long-range white-matter tracts connecting together the parieto-frontal areas of this network in the antero-poterior direction mature slowly during childhood, compared with transcallosal tracts that support functional connectivity between homotopic regions [42]. Such a grey and white-matter heterochronous maturation make these structures more fragile during brain development and could account for the expression of a medial parieto-frontal trait-pattern in patients vulnerable to schizophrenia, independently of the potential future emergence of acute psychotic episodes.

On the other hand, anatomo-functional changes in the TPJ, frequently reported in schizophrenia [43], [44], have been shown linked to the presence of particular symptoms. First, different sulcal displacements of this structure were evidenced in patients with inner and outer-space hallucinations [45]. Second, TPJ volume-reductions in schizophrenia were correlated with the severity of symptoms [46] or increased in patients who reported delusions of passivity [47]. Third, functional disturbances of this region were also correlated with the presence of psychotic symptoms [2], [24]. In our study, the correlation analysis results between the BOLD signal amplitude and the intensity of positive symptoms (Figure 2) fully support the idea that hyperactivity in the right IPL could be considered a ‘state-marker’ for acute psychotic episodes. In such a perspective, the IPL may play a critical role in deciphering the origin of a stimulus, since the more the information is ambiguous, the more the IPL is functionally recruited. Increased activity measured within this structure in patients with schizophrenia compared with controls during the ‘self’ condition, might result from specific difficulties on the part of the patients in eliminating the ambiguity of the signals when the representations are self-generated. Therefore, it seems reasonable to assume that schizophrenic patients may have a tendency to experience self abnormally, even without having to explicitly judge whether the stimulus is from self, or not. It could be hypothesized that severe positive symptomatology would result in complete overlap between the self and non-self cortical maps at the level of the IPL. This is exactly what we found in a recent case-study of a child suffering from early-onset schizophrenia [48] using the same self-other fMRI contrast-analysis as in our current study. Using repetitive Transcranial Magnetic Stimulation (rTMS) over the child's right IPL allowed increasing source-monitoring and agency performances, as well as normalizing the activity pattern in the cortical site. Interestingly, studies of virtual lesions in healthy volunteers examining the exact opposite to this case-report, confirmed that the participants made more self false-alarms after TMS was applied over the right IPL, than when it was applied to a control contra-lateral site [49].

In order to test whether externally-generated stimuli would rely on a low-level impairment in schizophrenia, we used an implicit procedure. As a consequence, this procedure did not allow for task-performance monitoring within the scanner. It seems therefore essential to exclude alternative explanations of the results which could be due for instance to a poorer performance by the patients or to the fact they might be more likely than controls to repeat someone else's voice within their head. Our results allow us to think that such interpretations are unlikely. First due to its simplicity the task itself did not result in significant differences in the behavioral performances between the two groups. Indeed, control tasks implemented outside the scanner confirmed that patients with schizophrenia performed the task similarly to healthy subjects. Secondly, the activation patterns measured in the other vs. self contrast (<EGV> - <SGV>) confirmed that participants from the both group performed the task adequately within the scanner (i.e. covert speak during <EGV> and passively listen during <EGV>). Moreover assuming a greater tendency to action (i.e. repeat voices) in the patients seems improbable since no group difference was evidenced within premotor and motor areas in this contrast analysis. For all these reasons, we believe that our fMRI procedure provides strong evidences for an increased overlap between the neural representations for self and others in schizophrenia.

Even though self-awareness impairment has already been suggested in schizophrenia, our study is the first evidence of neural impairments during implicit self-other distinction, in accordance with recent behavioural data [50]. Within this network, the anterior and posterior cingulate cortices were not shown to have a proportional relationship with the severity of positive symptoms. This alteration could therefore constitute a potential bio-marker for the disease. The recent research program for editing the DSM-V classification clearly emphasized the need to apply this sort of neuroscience findings, which reflect pathophysiological processes, to help with screening and earlier diagnosis of psychiatric disorders [51]. However, the activity measured in the right IPL correlated strongly with the current status of the patients, and so should perhaps be considered more as a state-marker for FRS. The right IPL could in fact prove to be a possible target for dimensional treatment, such as rTMS. Finally, in order to distinguish between the different specific FRS, which are linked with the feeling of agency, further studies which replicate our paradigm and include different sub-groups, such as hallucinators vs. non-hallucinators, and healthy subjects with a high genetic risk of developing schizophrenia are required.
